# Repurposing Histaminergic Drugs in Multiple Sclerosis

**DOI:** 10.3390/ijms23116347

**Published:** 2022-06-06

**Authors:** Susanna Amadio, Federica Conte, Giorgia Esposito, Giulia Fiscon, Paola Paci, Cinzia Volonté

**Affiliations:** 1 IRCCS Santa Lucia Foundation, Preclinical Neuroscience, Via del Fosso di Fiorano 65, 00143 Rome, Italy; s.amadio@hsantalucia.it (S.A.); gesposit@sissa.it (G.E.); 2 Institute for Systems Analysis and Computer Science “A. Ruberti” (IASI), National Research Council (CNR), Via dei Taurini 19, 00185 Rome, Italy; federica.conte@iasi.cnr.it (F.C.); fiscon@diag.uniroma1.it (G.F.); paci@diag.uniroma1.it (P.P.); 3 Department of Computer, Control, and Management Engineering “A. Ruberti” (DIAG), Sapienza University of Rome, 00185 Rome, Italy

**Keywords:** amodiaquine, diphenhydramine, H1 receptor, histamine N-methyltransferase, multiple sclerosis, network medicine, rupatadine

## Abstract

Multiple sclerosis is an autoimmune disease with a strong neuroinflammatory component that contributes to severe demyelination, neurodegeneration and lesions formation in white and grey matter of the spinal cord and brain. Increasing attention is being paid to the signaling of the biogenic amine histamine in the context of several pathological conditions. In multiple sclerosis, histamine regulates the differentiation of oligodendrocyte precursors, reduces demyelination, and improves the remyelination process. However, the concomitant activation of histamine H1–H4 receptors can sustain either damaging or favorable effects, depending on the specifically activated receptor subtype/s, the timing of receptor engagement, and the central versus peripheral target district. Conventional drug development has failed so far to identify curative drugs for multiple sclerosis, thus causing a severe delay in therapeutic options available to patients. In this perspective, drug repurposing offers an exciting and complementary alternative for rapidly approving some medicines already approved for other indications. In the present work, we have adopted a new network-medicine-based algorithm for drug repurposing called SAveRUNNER, for quantifying the interplay between multiple sclerosis-associated genes and drug targets in the human interactome. We have identified new histamine drug-disease associations and predicted off-label novel use of the histaminergic drugs amodiaquine, rupatadine, and diphenhydramine among others, for multiple sclerosis. Our work suggests that selected histamine-related molecules might get to the root causes of multiple sclerosis and emerge as new potential therapeutic strategies for the disease.

## 1. Introduction

Multiple sclerosis (MS) is a T-cell mediated autoimmune disease characterized by a strong inflammatory reaction that builds up into the CNS by the accumulation of inflammatory infiltrates and macrophage/microglia [1]. These molecular and cellular events contribute to severe demyelination, consequent neurodegeneration, and formation of lesions, mostly in the white matter of the optic nerve, brain stem, basal ganglia, spinal cord, and grey matter of the cerebral cortex [2]. The location of these lesions within the CNS characterizes the neurological symptoms of MS that include autonomic, visual, motor, and sensory impairments. Whereas the causes of the disease are not defined yet, MS occurs as a combination of genetic variations, environmental factors, viral infections, overall leading to key pathogenic mechanisms comprising direct damage/loss of myelin-producing cells caused by immune system deregulation [3,4]. While several diagnostic approaches and new therapeutic solutions are under investigation, up to now there is no curative but only palliative treatment for the various, either not predictable clinically isolated syndrome, or relapsing-remitting, or primary/secondary progressive forms of the disease [5].

Currently, increasing attention is paid to the signaling of the biogenic amine histamine in the context of several pathological conditions such as Parkinson’s disease [6], sleep disorders, schizophrenia, cerebral ischemia [7], amyotrophic lateral sclerosis (ALS) [8], Alzheimer’s disease [9] or physiological conditions of the CNS, such as memory [10,11] and neurodevelopment [12]. Regarding MS, it is well established that histamine is directly involved in the disease, for instance by regulating the differentiation of oligodendrocyte precursors, reducing demyelination, and improving the remyelination process [13,14,15,16,17]. However, the concomitant activation of histamine H1–H4 receptors can sustain either damaging or beneficial effects in MS, depending on the specific receptor subtype/s that become/s activated, the timing of receptor engagement, and the central versus peripheral target district that is involved [14,18]. For instance, the levels of histamine were found to be lower in the serum of relapsing-remitting MS patients compared to healthy individuals [19], and a recent study showed that the histamine precursor histidine is also lower in the serum of MS women with disabling and persistently perceived fatigue, thus suggesting a strong involvement of histamine in MS-associated symptoms too [20]. Nevertheless, in MS patients the levels of histamine in the CNS show an opposite trend with respect to the serum, an issue thus requiring further investigations [21,22].

Conventional drug discovery and development has failed so far to pour in the pipeline curative drugs for MS, causing a severe delay in the effective therapeutic options now available to attending physicians. In this perspective, drug repurposing offers an exciting and complementary alternative to rapidly approve some medicines already approved for other indications [23,24]. To identify novel drug-repurposing opportunities in the pursuit of unconventional but more efficacious MS treatments, promising insights come from the emerging field of system network theory and its application to medicine, known as network medicine [25,26,27]. As computational methods evolve, network medicine increases its capability of capturing the genetic and molecular intricacy of human diseases, and dissecting how such complexity rules disease manifestations, prognosis and, importantly, therapy [28,29,30].

In the present study, we adopted a new network-medicine-based algorithm for drug repurposing called SAveRUNNER [31,32] that has already shown good applicability to a neuroinflammatory/neurodegenerative disease such as ALS [33]. By quantifying the interplay between MS-associated genes and drug targets in the human interactome, and by exploiting a gene set enrichment analysis (GSEA), we identified new drug-disease associations and predicted off-label novel use of drugs for MS. Our work shows that selected histamine-related molecules might get to the root causes of MS and emerge as new potential therapeutic strategies for the disease.

## 2. Materials and Methods

### 2.1. Data Retrival

The human interactome (i.e., the cellular network of all physical molecular interactions), was downloaded from Cheng and co-authors [28], where the authors assembled their in-house systematic human protein–protein interactome with 15 commonly used databases of several types of experimental evidence (e.g., binary PPIs from three-dimensional protein structures; literature-curated PPIs identified by affinity purification followed by mass spectrometry, Y2H, and/or literature-derived low-throughput experiments such as BioGRID [34], HPRD [35], MINT [36], IntAct [37], InnateDB [38]; signaling networks from literature-derived low-throughput experiments; kinase-substrate interactions from literature-derived low-throughput and high-throughput experiments). This combined version of the interactome is composed of 217,160 protein–protein interactions connecting 15,970 unique proteins.

Disease-associated genes were downloaded from Phenopedia [39], which collects gene associations for 3255 diseases (released 27 April 2020).

Drug-target interactions were acquired from DrugBank [40], which contains 13,563 drug entries, including 2627 approved small molecule drugs, 1373 approved biologics, 131 nutraceuticals, and over 6370 experimental drugs (released 22 April 2020). The targets Uniprot IDs provided by DrugBank were mapped to Entrez gene IDs by using the BioMart-Ensembl tool (https://www.ensembl.org/, accessed on 22 April 2020). For some drugs of interest for which no targets were found in DrugBank, we integrated drug-target interactions available from the Therapeutic Target Database [41].

### 2.2. SAveRUNNER Algorithm

We recently developed SAveRUNNER [31,32], a new network-medicine-based algorithm for drug repurposing, with the aim of identifying new potential indications for currently marketed drugs and improving the efficacy of experimental validation. The SAveRUNNER algorithm has been already and successfully applied to several diseases, including amyotrophic lateral sclerosis [33]. A detailed description of the SAveRUNNER algorithm is reported in [31].

### 2.3. Gene Set Enrichment Analysis

In order to verify whether the candidate anti-MS repurposable drugs predicted by SAveRUNNER could counteract the gene expression perturbations caused by the MS pathophenotype (i.e., if they can up-regulate genes down-regulated by the disease or vice versa), we performed a gene set enrichment analysis (GSEA). We first collected three gene expression datasets of MS patients and control samples available through the GEO public repository (i.e., GSE38010, GSE26927, GSE32645). For each dataset, we exploited the GEO2R interactive web tool that makes use of limma package to identify genes that are differentially expressed across the two analyzed experimental conditions (i.e., control versus MS samples). The obtained *p*-values were adjusted for multiple hypotheses testing, by using Benjamini–Hochberg procedure. In order to select statistically significant differentially expressed genes, we set a threshold on the adjusted *p*-values equal to 0.05. We used the obtained three lists of differentially expressed genes as three MS disease signatures. Then, we queried the Connectivity Map (CMap) database that collects high-throughput reduced representation gene expression data obtained by using L1000 assay [42,43]. A total of 27,927 perturbagens were profiled in a core set of 9 cell lines to produce 476,251 expression signatures. We used the differentially expressed genes of drug-treated human cell lines from the CMap database as drug signatures.

For each drug that was present in both the CMap database and predicted by SAveRUNNER to be effective against MS, we evaluated the treatment effects on differentially expressed genes obtained for MS disease phenotype, by exploiting the CMap query tool for each MS signature given as a separated input list [43]. A selected repurposing candidate drug was considered to have a potential treatment effect against MS if the drug signature was negatively correlated with the MS signature. We stated that drugs and diseases were negatively correlated if the corresponding CMap computed score was negative, and we assigned a score equal to 1 to that drug for that MS signature. Following the procedure adopted in [44], the number of MS datasets satisfying this criterion was used for each drug as the final GSEA score, which ranges from 0 (not available) to N, being N the total number of MS analyzed datasets. By considering in this study N = 3 disease signatures, the maximum GSEA score for each drug was 3.

### 2.4. Human Brain Tissue

Frontal cortex tissue was collected post mortem by UK Multiple Sclerosis Tissue Bank at Imperial College, London. Demographic and clinical characteristics of MS cases at the time of tissue collection are reported (Table 1). Frontal cortex tissues are from 5 neuropathological confirmed cases of MS, matched for disease courses (all secondary progressive MS, SPMS) but presenting different ages (range 45–76 years), sex, disease durations (range 6–36 years), causes of death, and death-tissue preservation intervals expressed in hours (DTPI). Analysis also was performed on samples from 2 patients who died by non-neurological diseases. Cerebral hemispheres were fixed with 4% paraformaldehyde for 2 weeks, coronally sliced, and blocked. Individual blocks were cryoprotected in 30% sucrose for 1 week, frozen in isopentane and stored at –80 °C until use.

### 2.5. Immunohistochemistry

Immunohistochemistry was performed as described [45,46]. Human sections (30–40 μm) were pre-incubated for 10 min with 5% H_2_O_2_ in 5% methanol in PBS, and for 24–48 h in PBS-0.3% Triton X-100, 2% normal donkey serum at 4 °C, with H1 receptor polyclonal antiserum (1:200, Santa Cruz Biotechnology, Dallas, TX, USA) or HNMT polyclonal antiserum (1:200, Atlas Antibodies, Bromma, Sweden). Sections were then incubated with biotinylated donkey anti-rabbit antibodies (Jackson ImmunoResearch Europe Ltd., Suffolk, UK), followed by avidin-biotin-peroxidase reactions (Vectastain, ABC kit, Vector, Burlingame, CA, USA), using 3,3′-diaminobenzidine (Sigma-Aldrich, Saint Louis, MO, USA) as a chromogen. Sections were mounted on poly-lysine slides and air dried for 24 h. The histological preparations were examined using an Axioskop 2 light microscope (Zeiss, Oberkochen, Germany). Images were taken with Neurolucida software (MBF Bioscience, Williston, VT, USA).

### 2.6. Immunofluorescence

Human sections (30–40 μm) were blocked with 10% normal donkey serum in 0.3% Triton X-100 in PBS and incubated with HNMT polyclonal antiserum (1:200, Atlas Antibodies) and with GFAP (glial fibrillary acidic protein, 1:500 Novus Biological Centennial, CO, USA) antibody, marker for astrocytes, in 0.3% Triton X-100 and 2% normal donkey serum in PBS, for 48 h at 4 °C and processed for double immunofluorescence. The secondary antibodies in 0.3% Triton X-100 and 2% normal donkey serum in PBS were Alexa Fluor^®^ 488-AffiniPure donkey anti-rabbit IgG (1:200, Jackson Immunoresearch, green) and Cy3-conjugated donkey anti-mouse IgG (1:100, Jackson Immunoresearch, red).

### 2.7. Confocal Microscopy

Immunofluorescence analysis was performed by confocal laser scanning microscope (Zeiss, LSM800) equipped with four laser lines: 405, 488, 561, and 639 nm. Brightness and contrast were adjusted with Zen software (Zeiss).

### 2.8. Protein Extraction and Western Blotting

Six different snap-frozen blocks of frontal cortex from 3 independent SPMS cases and one block from 6 different control cases were processed for protein extraction. Detergent-soluble proteins were extracted with Ripa buffer (1% Nonidet P-40, 0.5% sodium deoxycholate, 0.1% SDS in PBS, containing protease inhibitors), using a micropestle. After a short sonication, the homogenates were incubated on ice for 1 h and centrifuged at 12,000 rpm for 10 min at 4 °C. Protein quantification was performed from the supernatants by Bradford colorimetric assay (Biorad, Milan, Italy). Proteins (30 μg) were separated by gel electrophoresis on 10% SDS-PAGE and transferred to nitrocellulose Hybond-C-extra membranes (Amersham Biosciences, Buckinghamshire, UK). Filters were pre-wetted in 5% blocking agent in TBS-T (10 mM Tris pH 8, 150 mM NaCl, 0.1% Tween 20) and hybridized overnight with H1 receptor polyclonal antiserum (1:500, Santa Cruz) or HNMT polyclonal antiserum (1:400, Atlas Antibodies). Signals were detected with anti-rabbit horseradish peroxidase-conjugated antibody (1:5000) and developed by enhanced chemiluminescence (Amersham Biosciences) using Kodak Image Station (KDS IS440CF). Semi-quantitative analysis was performed with Image J software.

### 2.9. Statistical Analysis

Data are presented as means ± standard error of the means (SEM), and statistical analysis was determined by ANOVA. Statistical difference between groups was verified by Student’s *t*-test with * *p* < 0.05 considered as statistically significant.

## 3. Results

### 3.1. MS-Drug Network

In the present study, we exploited the recently developed SAveRUNNER algorithm [31,32] for the identification of novel repurposable drug candidates for MS. SAveRUNNER identified 540 repurposable drugs (out of 1873 U.S. Food and Drug Administration-approved drugs) that are significantly associated (*p*-value < 0.05) with MS (Supplementary Table S1). Supplementary Table S1 (sheet 1) lists all the identified drug candidates in descending order of network-based similarity values (from 1 to 0), GSEA scores (from 3 to 0), and alphabetical order, thus prioritizing the candidates most eligible in satisfying appropriate and probable MS repositioning.

Among the 540 identified drugs, we first highlight the second compound of the list, the orally active 4-aminoquinoline derivative, amodiaquine (IUPAC name 4-[(7-chloroquinolin-4-yl)amino]-2-(diethylaminomethyl)phenol; DrugBank accession number DB00613; other brand names Basoquin, Camoquin, Flavoquine; current indication for treatment of acute malarial attacks in non-immune subjects), which exhibits a network-based similarity value = 1, and *p*-value = 0.0080, thus appearing as a potential very good candidate to be further exploited in the context of MS.

Amodiaquine is a drug possessing anti-inflammatory properties and inhibiting histamine N-methyltransferase (HNMT), one of the two enzymes involved in the catabolism of histamine in mammals, and the sole enzyme catabolizing histamine in the CNS. HNMT is present in most body tissues but not in serum. Both peripheral (intraperitoneal) and central (intraventricular) administration of amodiaquine substantially reduces HNMT activity [47]. Consistently, amodiaquine is reported to boost the amount of histamine in cells and tissues, and recent studies suggest that an increase in brain histamine levels by HNMT inhibition could contribute to the improvement of brain disorders [48], although the impact of amodiaquine on specific brain functions is still not fully investigated.

**Figure 1:**
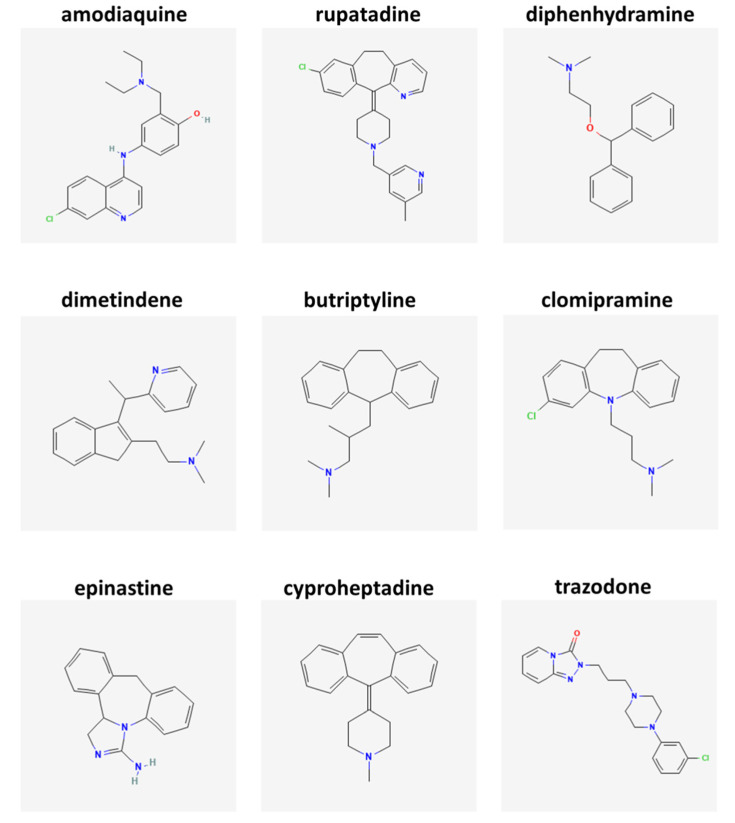
Chemical structures of histamine candidate drugs repurposable for multiple sclerosis. The figure shows the chemical structures of the best selected histaminergic modulators that are identified by SAveRUNNER/GSEA algorithm as repurposable drugs for multiple sclerosis. 2D chemical structures were retrieved from https://pubchem.ncbi.nlm.nih.gov/ (accessed on 12 May 2022).

Among additional MS-repurposable drugs prioritized by SAveRUNNER and classified as histaminergic drugs, of particular note is rupatadine (DrugBank accession number DB11614; brand name Rupall; a dual histamine H1 receptor and platelet activating factor receptor antagonist that is used for symptomatic relief in seasonal and perennial rhinitis and chronic spontaneous urticaria), with network-based similarity value = 0.6279, and *p*-value = 0.0141, ranking in position 243 of the list. Shortly thereafter (position 251), we found diphenhydramine (DrugBank accession number DB01075; brand name Benadryl; a first generation H1 receptor antihistamine with low antagonist affinity at muscarinic acetylcholine M2 receptor, used in the treatment of seasonal allergies), showing network-based similarity value = 0.6279, and *p*-value = 0.0157. Next (position 276), we found dimetindene (DrugBank accession number DB08801; brand names Fenistil, Foristal, Vibrocil; a selective H1 receptor antagonist and weak muscarinic acetylcholine M2 receptor antagonist, used for symptomatic treatment of allergic reactions), possessing network-based similarity value = 0.6279, and *p*-value = 0.0165. With network-based similarity values between 0.5038 (butriptyline, position 348) and 0.0198 (amoxapine, position 539), we next identified 41 additional drug candidates that all share antagonistic actions at histamine H1 receptor (Supplementary Table S1, sheet 2), including clomipramine (position 411), epinastine (position 417), cyproheptadine (position 427), and trazodone (position 428). These findings strongly corroborate our hypothesis that modulators of H1 signaling might be further considered as therapeutically exploitable for MS (Table 2). The chemical structures of these drugs are reported in Figure 1.

### 3.2. Gene Set Enrichment Analysis of Anti-MS Repurposable Drugs

To further investigate the anti-MS repurposable drugs predicted by SAveRUNNER, we performed GSEA analysis by using transcriptome data from nervous system tissues of MS patients as disease signatures, and gene expression data of drug-treated human cell lines from the Connectivity Map (CMap) database as drug signatures. For each drug predicted by SAveRUNNER and included in the CMap database, we computed a GSEA score as an indication of its possible counteraction of gene expression perturbations caused by MS pathophenotype. In particular, for each MS dataset, we selected drugs whose signatures were negatively correlated with the MS signature according to the CMap query tool [42,43,49], as able to have a potential treatment effect against differentially expressed genes in MS samples (see Materials and Methods). The assigned GSEA scores, ranging from 1 to 3, correspond to the number of MS datasets satisfying this criterion for a specific drug. According to GSEA analysis, we found 195 drugs with GSEA score equal to 3, including the HNMT inhibitor amodiaquine, and the H1 receptor antagonists rupatadine, clomipramine cyproheptadine, and trazodone, 49 compounds with GSEA score equal to 2, including the H1 antagonists diphenhydramine, epinastine, and amitriptyline (position 513, network-based similarity value = 0.1395, and *p*-value = 0.0034), and 14 with GSEA score equal to 1 (Table 2 and Supplementary Table S1, sheet 1).

**Figure 2:**
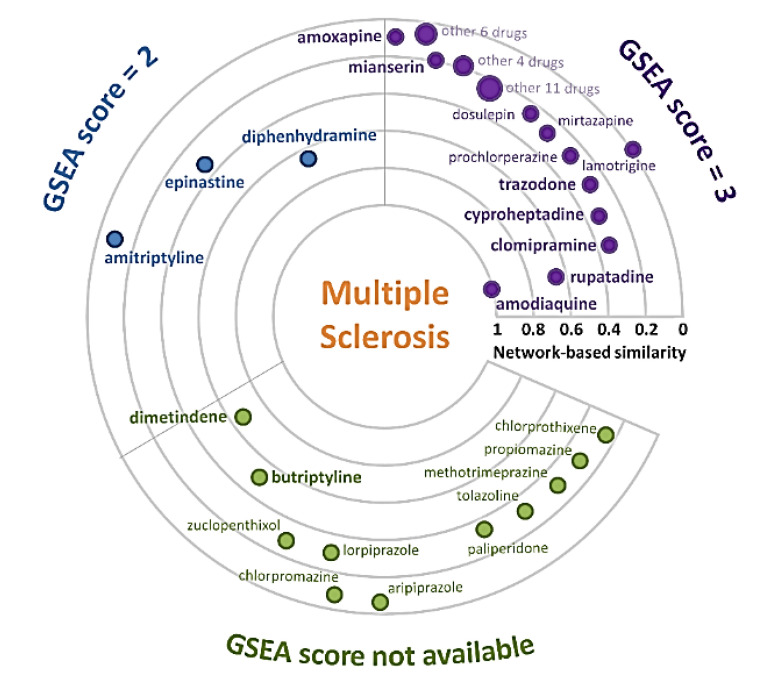
Histamine candidate drugs repurposable for multiple sclerosis. Radial plot reporting the network-based similarity measure(s) between multiple sclerosis (MS) and the candidate drugs belonging to the histamine class and predicted by SAveRUNNER as repurposable for MS. Each drug is represented as a circle. Groups of drugs with the same values of similarity measure are indicated with increasing circle size. The farther a drug is from the center (or closer to), the more distant (or proximal) the drug-targets are from the MS-associated genes in the human interactome. Each drug is colored according to the Gene Set Enrichment Analysis (GSEA) score.

From the combined SAveRUNNER/GSEA analysis (Figure 2), we evince that amodiaquine, rupatadine, and diphenhydramine are the best histaminergic drug-repurposing candidates, with network-based similarity values between 1 and 0.6279, and GSEA scores equal to 3 or 2 (Table 2 and Supplementary Table S1, sheet 2), while, for instance, dimetindene and butriptyline have a GSEA score not available, although possessing quite high network-based similarity values in the range 0.6–0.5 (Table 2 and Supplementary Table S1, sheet 2).

### 3.3. HNMT Is Present on Astrocytes in the Parenchyma of Secondary Progressive MS Frontal Cortex

**Figure 3:**
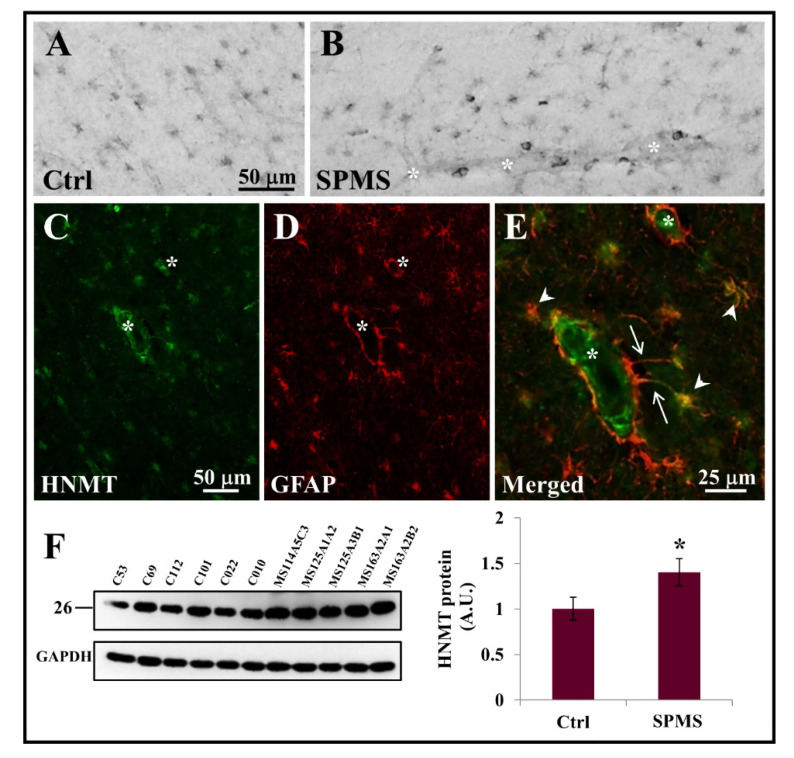
Histamine N-methyltransferase expression in human cortical white matter. Human cortical sections (30–40 µm) from healthy subjects (Ctrl) and secondary progressive multiple sclerosis patients (SPMS) were subjected to immunohistochemistry for histamine N-methyltransferase (HNMT) (**A**,**B**), or to double immunofluorescence confocal analysis for HNMT and glial fibrillary acidic protein (GFAP) (**C**–**E**). Capillary blood vessel in panels (**B**–**E**) are highlighted by white asterisks. HNMT protein content in Ctrl and SPMS cortical tissue (30 µg extract) was analyzed by Western blotting (**F**), * *p* < 0.05.

In the CNS, histamine is almost exclusively catabolized to the selective H3 receptor agonist N-alpha-methylhistamine by HNMT [48,50,51], since the other histamine degrading enzyme diamine oxidase (DAO) is reported not to be expressed in the CNS [52]. In this way, the central neurotransmitter activity of histamine is rather exclusively regulated by HNMT, whose localization in the nervous system becomes crucial during disease conditions. After our SAveRUNNER/GSEA analysis identified the HNMT inhibitor amodiaquine as one of the best MS repurposable drug candidates, we investigated the HNMT protein distribution in the parenchyma of frontal cortex from 5 neuropathological confirmed cases of secondary progressive multiple sclerosis (SPMS) and 2 patients who died by non-neurological diseases (Table 1). By immunohistochemistry, a clear HNMT signal was detected in the soma and fibers of distinct cell types scattered throughout white and grey matter of frontal cortex tissue (Figure 3A), appearing particularly enriched in the vicinity of capillary blood vessels (Figure 3B–E, white asterisks).

Major differences of expression between control and SPMS tissues were not apparently detected by immunohistochemistry or immunofluorescence analysis (data not shown). Double immunofluorescence confocal examination indicated the absence of co-localization of HNMT with MHCII marker of macrophages/microglia (data not shown). HNMT signal (green color) was instead strongly expressed in glial fibrillary acidic protein (GFAP)-positive astrocytes (red color) found in both grey (data not shown) and white matter of both control (data not shown) and SPMS patients (Figure 3C–E). In the vicinity of blood vessels (white asterisks), a strong HNMT signal was particularly evident in fibrous astrocytes (arrowheads, Figure 3E) exhibiting unbranched cellular processes (arrows) often protruding “vascular feet” that are physically connected to the external capillary wall [53] (Figure 3E, yellow merged signal). By immunohistochemistry, we also detected the presence of HNMT signal on neuronal cell bodies and axons present in frontal cortex grey matter (data not shown).

To investigate if disease progression modifies HNMT protein content, we analyzed total cell extracts from SPMS and control frontal cortex by Western blotting. A statistically significant increase of HNMT protein as a single specific protein band with estimated molecular mass in the 25–27 KDa range was observed in tissue from SPMS patients, compared to healthy subjects (Figure 3F).

### 3.4. Expression of Histamine H1 Receptor in Frontal Cortex

**Figure 4:**
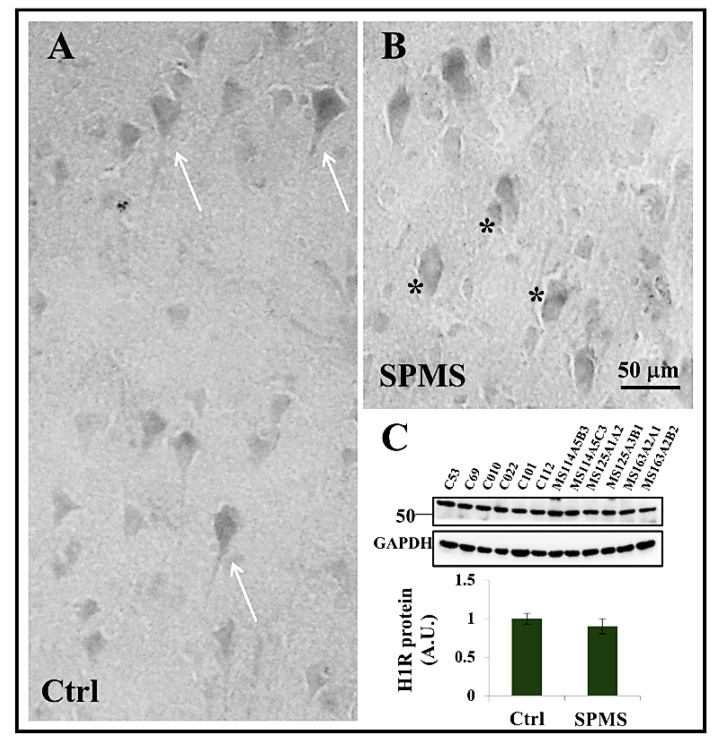
H1 receptor expression in human cortical grey matter. Human cortical sections (30–40 µm) from healthy subjects (Ctrl) and secondary progressive multiple sclerosis patients (SPMS) were subjected to immunohistochemistry for H1 receptor (**A**,**B**). Damaged and smaller neurons in panel (**B**) are highlighted by black asterisks. H1 protein content in Ctrl and SPMS cortical tissue (30 µg extract) was analyzed by Western blotting (**C**).

Since the SAveRUNNER/GSEA drug candidates rupatadine, diphenhydramine, dimetindene, butriptyline, clomipramine, epinastine, cyproheptadine, and trazodone share antagonistic actions at histamine H1 receptor, by immunohistochemistry we analyzed the presence of this receptor in the parenchyma of frontal cortex from 5 neuropathological confirmed cases of SPMS and 2 patients who died by non-neurological diseases (Table 1). H1 signal was distinctly notable in both control (Figure 4A) and SPMS (Figure 4B) grey matter parenchyma, highlighting the cell soma and axons of pyramidal neurons (Figure 4A, arrows) that appear damaged and smaller in shape in SPMS tissue (Figure 4B, black asterisks). We could not detect the presence of H1 receptor signal in astrocytes scattered throughout the grey or white matter of cortical parenchyma (data not shown), thus confirming previous results about the lack of this receptor in inactive astrogliotic plaques of MS patients [54].

Moreover, by Western blot analysis of frontal cortex extracts, we distinguished H1 receptor as a single specific protein band with estimated molecular mass in the 50–55 KDa range (Figure 4C) and whose protein content was not statistically modulated in SPMS patients compared to control.

## 4. Discussion

Network theory proposes a manageable structure for refining relevant insights from the emerging sets of molecular omics data. Network medicine embraces this concept and rather than forcing disease pathogenesis into a reductionist model, it acknowledges the complexity of multiple influences on disease, viewed as complex network of interconnected molecular determinants. To understand the relation between perturbations on the molecular level and phenotypic disease manifestations, network medicine relies on various types of networks, in which different molecular entities such as genes, proteins, metabolites, or disease-phenotypes, and drugs (defined as “nodes”), are linked according to their mutual interconnections (defined as “links”), for instance represented by physical interactions, transcriptional induction/inhibition, enzymatic activation, shared signaling, distinct disease manifestations. Understanding the effect of these interconnections on diseases onset and progression could thus lead to the identification of new therapeutic strategies.

In the present work, we exploited a network medicine-based algorithm SAveRUNNER [32], complemented with a GSEA analysis, to prioritize some predicted drugs according to their network-based similarity with MS, and identified interesting and novel not customary potential MS drugs belonging to the histaminergic modulation system. By analyzing their chemical structures, we noticed that five out of the nine best H1 receptor antagonists were azepine derivatives sharing unsaturated heterocyclic rings of seven atoms, some of which had a nitrogen replacing a carbon. While the relationship between structure and function is widely recognized as a central and crosscutting concept in pharmacology and medical science, it is quite difficult at this point to establish if the positive structure–activity correlation of rupatadine, butriptyline, clomipramine, epinastine, and cyproheptadine indeed contributes to their prioritization as best repurposable drug candidates for MS. Further studies might shed light on this specific issue.

Among the best compounds emerging from the SAveRUNNER/GSEA analysis, we found an inhibitor of the histamine degrading enzyme HNMT, amodiaquine, known for more than 40 years as histamine content regulator and anti-inflammatory agent. This result corroborates previous data that indeed established an altered metabolism of histamine during MS, perhaps associated to immune reactions, although to date the beneficial versus detrimental role of peripheral/central histamine in MS pathogenesis has not been conclusively established. For instance, the assessment of histamine concentration (as well as of the diamine oxidase enzyme involved in the oxidation and inactivation of histamine) in blood serum has shown significant lower levels in relapsing-remitting MS patients with respect to healthy subjects [19]. In the cerebrospinal fluid (CSF), the histamine levels were instead reported to be about 60% higher in patients with either relapsing-remitting or progressive forms of MS with respect to healthy individuals. Coherently, HNMT activity in CSF is significantly lower in both groups of MS patients [55], and more recently Kallweit and colleagues confirmed significant elevation of histamine levels in MS CSF [56]. However, this issue is still controversial, since previous work reported that histamine and the HNMT metabolite N-alpha-methylhistamine are instead constant in MS compared to control CSF [57].

By looking at the only enzyme responsible for histamine catabolism in the CNS, HNMT, our protein expression results obtained in cortical tissue now indicate that the enzyme is mainly expressed on astrocytes and up-regulated in MS. Astrocytes play an important role in the lesion formation during MS [58]. In acute lesions, astrocytes display a proliferative and hypertrophic response, and produce various neurotrophic factors and cytokines, or remove cytotoxic factors, thus contributing to remyelination and axonal protection. However, the subsequent formation of a glial scar represents a major mechanical impediment to remyelination and axonal regeneration. Based on this knowledge, our hypothesis would be that HNMT enriched on astrocytes might cause depletion of histamine in MS cortical white matter and thus play a detrimental role by promoting the glial scar and impeding histamine-promoted remyelination [13,59,60]. There is current growing interest in better understanding the etiology of astrogliosis and the mechanisms by which the glial scar can be reduced. In this perspective, the HNMT inhibitor amodiaquine prioritized by SAveRUNNER/GSEA analysis might likely be used to restore a proper histamine balance and ameliorate MS conditions.

While the HNMT expression data that we presented would not exclude histamine as a possible mediator implicated in the pathogenesis of MS, the frequencies of HNMT genotypes and allelic variants do not differ significantly between MS patients and controls, and are also unrelated with the age of onset, gender, course, and risk for the disease [61]. However, a single nucleotide polymorphism of the HNMT gene on exon 4C314T of the chromosome 2q22.1 (causing the amino acid substitution Thr105Ile and decreasing enzyme activity) was investigated so far, thus rendering any correlation still premature. Differently from MS, for instance, ALS patients carrying the Ile105 polymorphism on the Thr105Ile allele in the HNMT gene exhibit a trend toward a significant delay in symptom onset [62].

Of note, amodiaquine is recognized not only as a potent and selective HNMT inhibitor [63] but also an anti-malarian [64], anti-inflammatory agent [65], and recently reported possessing anti-coronavirus activity [66]. Moreover, amodiaquine reduces microgliosis, neuronal loss, impairment of adult hippocampal neurogenesis, and deposition of Aβ plaques, overall leading to significant amelioration, for instance, of typical Alzheimer’s disease features in the 5XFAD mouse model [67]. However, anti-inflammatory actions are in part sustained by a concomitant agonistic action of amodiaquine on the orphan nuclear receptor Nurr1 [67,68]. This notion certainly opens a more realistic identification of amodiaquine as a versatile poly-functional molecule, a feature shared not only by several drugs, but also occurring in the context of various disease conditions. As such, amodiaquine can truly exemplify the multi-targeted therapeutic approach currently adopted for better studying and treating complex and heterogeneous diseases including MS.

Histamine has been previously shown to be implicated in several ways in the pathogenesis of MS and experimental autoimmune encephalomyelitis (EAE) MS model. For instance, EAE severity is augmented in histidine decarboxylase deficient mice unable to synthetize histamine, thus suggesting a major beneficial action of histamine in MS [69]. However, mice simultaneously depleted of H1, H2, H3, and H4 receptors and consequent histaminergic receptor signaling have demonstrated significant resistance to EAE [70]. On the other hand, histamine can sustain pro-inflammatory actions such as increased blood brain barrier permeability and immune cell infiltration into the CNS via H1 receptors, and these actions worsen the MS disease condition [13]. Moreover, there is overexpression of the H1 receptor gene in acute lesions of relapsing remitting MS patients [71], while H1 transcript is significantly lower in secondary-progressive-MS patients with respect to healthy individuals [72], indicating a distinct involvement of histamine receptors in the different forms of the disease. Substantiating our results about the presence of H1 protein in cortical parenchyma of MS patients, the SAveRUNNER/GSEA analysis showed that several H1 receptor antagonists, especially rupatadine and diphenhydramine (and to a lesser extent, dimetindene, butriptyline, clomipramine, epinastine, cyproheptadine, and trazodone), can be prioritized as novel drug candidates to be repurposed for MS by possessing relevant network-based similarity values and GSEA scores. The shared receptor binding properties of these compounds, moreover, confirm H1 receptor as an additional target to be exploited for therapeutic purposes in MS. Our results thus consolidate previous reports indicating that decreased EAE susceptibility is obtained in H1 receptor-depleted mice [73], while overexpression of H1 in T cells becomes disease-promoting [74]. The propathogenic role of H1 receptor is further confirmed in additional work demonstrating that H1 antagonists hydroxyzine or pyrilamine reduce disease severity and pathology in EAE rats and mice, respectively [75,76]. Hydroxyzine has demonstrated efficacy also in decreasing mood symptoms in a pilot open-label clinical trial with relapsing-remitting or relapsing-progressive MS patients [77]. Moreover, the reduced incidence of MS in patients exposed to sedating H1 antagonists further validates the involvement of this receptor in MS [78], thus strongly encouraging the use particularly of SAveRUNNER/GSEA best predicted rupatadine and diphenhydramine, along with amodiaquine as discussed above, as novel drugs to be soon validated for MS in preclinical work. Last but not least, H1 antagonists have shown also remyelinating properties for the treatment of chronic demyelinating injury in multiple sclerosis clinical trials [79].

However, suffering from the notorious incompleteness of literature-based input parameters, we have to be aware that SAveRUNNER/GSEA analysis may also lead to incomplete predictions. This could explain, for instance, the absence of the above-described hydroxyzine or pyrilamine from the list of MS drugs predicted by the SAveRUNNER/GSEA algorithm.

Another important issue that emerges from the present work is what we can further learn by applying the SAveRUNNER/GSEA algorithm to different diseases, such as MS (the present work) compared to ALS, for instance [33]. Although sharing certain aspects of neuroinflammation, demyelination, neurodegeneration and muscle atrophy, eliciting cellular damage primarily in brain, spinal cord, and muscles, and finally causing similar symptoms such as fatigue, muscle spasms, inability to move, and, last but not least, sharing the lack of an available cure, MS and ALS have more differences than similarities. This dichotomy is also evident from SAveRUNNER/GSEA analysis. Undoubtedly, the H1 receptor antagonist clomipramine that emerges as a good candidate to be repurposed for MS, with good similarity value and GSEA score, confirms its repurposable potential previously also demonstrated against ALS [33]. However, the H1 antagonists mianserin (network-based similarity value = 0.2234, *p*-value = 0.0109, GSEA score = 3) and amoxapine (network-based similarity value = 0.0198, *p*-value = 0.0338, GSEA score = 3) that are ranked quite low in the MS druggable scale were previously demonstrated to be very promising ALS repurposable drug candidates (with network-based similarity values of 0.92 and 0.89, respectively) [33]. Finally, modafinil, which was identified by SAveRUNNER/GSEA analysis with the highest score of network similarity among the SAveRUNNER-predicted drugs for ALS [33], is not even considered a druggable target for MS. This confirms not only the reliability but also the disease-selectivity of the network medicine approach implemented through our algorithm applied to MS.

After having discussed our results in view of current knowledge, we can surely conclude that a network-medicine approach to drug repurposing can precisely and significantly accelerate innovation and proficiency in the identification and development of novel drugs to be validated for alleviating for instance allergic phenomena, depression/sleep disturbances, and demyelination in MS patients.

## Figures and Tables

**Table 1:** Demographic and clinical characteristics of MS cases at the time of cortical tissue collection.

Case	Age (Years)	Sex	Clinical Diagnosis	Disease Duration (Years)	Cause of Death	DTPI (h)
MS074	64	F	SPMS	36	Gastrointestinal bleed/obstruction, aspiration pneumonia	7
MS076	49	F	SPMS	18	Chronic renal failure, heart disease	31
MS114	52	F	SPMS	15	Pneumonia, sepsis, pulmonary embolism	12
MS125	76	F	SPMS	31	MS	13
MS163	45	F	SPMS	6	Urinary tract infection, MS	28

**Table 2:** Multiple sclerosis-histaminergic repurposable drugs candidates.

DRUG	Similarity Value	*p*-Value	GSEA Score	Drug Bank Code	Target
Amodiaquine	1	0.00806	3	DB00613	HNMT
Rupatadine	0.62791	0.01412	3	DB11614	H1 receptor
Diphenhydramine	0.62791	0.01571	2	DB01075	H1 receptor
Dimetindene	0.62791	0.01654	na	DB08801	H1 receptor
Butriptyline	0.50388	0.02857	na	DB09016	H1 receptor
Clomipramine	0.37984	0.03232	3	DB01242	H1 receptor
Epinastine	0.37984	0.0044	2	DB00751	H1 receptor
Cyproheptadine	0.36213	0.01584	3	DB00434	H1 receptor
Trazodone	0.36213	0.03221	3	DB00656	H1 receptor

The table lists the best selected histaminergic modulators that are identified by SAveRUNNER/GSEA algorithm as repurposable drugs for multiple sclerosis. The DrugBank code number and known target of each ligand are indicated, as from https://www.drugbank.ca/drugs/ (accessed on 31 March 2022). na = not available GSEA score for that drug.

## Data Availability

All data generated during this study are included in this published article as Supplementary Material. SAveRUNNER code is open-source and available at https://github.com/sportingCode/SAveRUNNER (accessed on 5 June 2021) together with an exhaustive and well-documented user guide, including a detailed description of all R scripts and all input/output files through a working example of SAveRUNNER application to 15 diseases.

## Supplementary Materials

The following supporting information can be downloaded at: www.mdpi.com/article/10.3390/ijms23116347/s1.
